# The pandemic gap of respiratory viruses during the COVID-19 pandemic

**DOI:** 10.1128/mbio.03376-25

**Published:** 2025-12-10

**Authors:** Viviana Simon, Daniel Floda, Charles Gleason, Ana Silvia Gonzalez-Reiche, Alberto E. Paniz-Mondolfi, Emilia Mia Sordillo, Peter Palese, Harm van Bakel

**Affiliations:** 1 Department of Microbiology, Icahn School of Medicine at Mount Sinai 5925, New York, New York, USA; 2 Center for Vaccine Research and Pandemic Preparedness (C-VaRPP), Icahn School of Medicine at Mount Sinai 5925, New York, New York, USA; 3 Department of Pathology, Molecular and Cell Based Medicine, Icahn School of Medicine at Mount Sinai 5925, New York, New York, USA; 4 Division of Infectious Diseases, Department of Medicine, Icahn School of Medicine at Mount Sinai 5925, New York, New York, USA; 5 The Global Health and Emerging Pathogens Institute, Icahn School of Medicine at Mount Sinai 5925, New York, New York, USA; 6 Department of Genetics and Genomic Sciences, Icahn School of Medicine at Mount Sinai 5925, New York, New York, USA; 7 Department of Artificial Intelligence and Human Health, Icahn School of Medicine at Mount Sinai 5925, New York, New York, USA; 8 Icahn Genomics Institute, Icahn School of Medicine at Mount Sinai 5925, New York, New York, USA; Tsinghua University, Beijing, China

**Keywords:** respiratory viruses, surveillance, epidemiology, influenza viruses, SARS-CoV-2

## Abstract

Respiratory viruses typically exhibit seasonal patterns, posing ongoing public health challenges. The coronavirus disease 2019 pandemic altered these patterns dramatically, with many common respiratory viruses disappearing from circulation for extended periods. Here we analyzed over three million diagnostic tests from a metropolitan healthcare system in New York City over 7 years, tracking severe acute respiratory syndrome coronavirus 2 (SARS-CoV-2) and eight common respiratory viruses before and during the pandemic. Following the initial SARS-CoV-2 wave in the spring of 2020, influenza A/B, respiratory syncytial virus, seasonal coronaviruses, parainfluenza, and human metapneumoviruses were absent for months to years, a phenomenon that we termed the “pandemic gap.” This disruption likely resulted from public health measures and SARS-CoV-2-induced antiviral immune responses resembling trained immunity. These findings suggest that the pandemic has temporarily reshaped respiratory virus epidemiology, potentially affecting immune development and increasing susceptibility to future respiratory virus epidemics.

**IMPORTANCE:**

In this retrospective study using millions of diagnostic tests over 7 years from patients at the Mount Sinai Health System in New York City, we show that when the coronavirus disease 2019 pandemic began in early 2020, many but not all common respiratory viruses disappeared from circulation. We observed prolonged absences ranging from 10 months to nearly 3 years for viruses such as influenza A/B viruses, respiratory syncytial viruses, seasonal coronaviruses, parainfluenza, and human metapneumoviruses. This unusual decline in enveloped respiratory RNA virus activities may have been linked to public health interventions like social distancing, wearing of masks, and lockdowns. Additionally, the rapid spread of severe acute respiratory syndrome coronavirus 2 may have triggered broad, pathogen-agnostic immune responses and the imprinting of antiviral signatures in innate immune cells that conferred temporary protection against other viruses. This phenomenon resembles “trained immunity,” a form of enhanced innate immune memory observed after certain infections or vaccinations.

## OBSERVATION

Respiratory viruses pose a significant threat to public health, with a high burden on healthcare systems and a constant global health risk (1–3). The circulation of many respiratory viruses is characterized by seasonality in the temperate regions of the Northern and Southern hemispheres (e.g., influenza viruses and respiratory syncytial virus [RSV] [1–3]). The outbreak of the 2020 severe acute respiratory syndrome coronavirus 2 (SARS-CoV-2) pandemic affected the seasonal pattern of different respiratory viruses in an unprecedented manner (4–6).

The Mount Sinai Health System (MSHS) is one of the largest health care providers of the New York City (NYC) metropolitan area, which encompasses regions from New York, Connecticut, and New Jersey. NYC itself has about 8.1 million people (18–23 million in the larger metropolitan area), making Greater New York the most densely populated metropolitan area in the United States. It is a confluence of diverse human populations with a broad range of underlying medical conditions and is highly reliant on public transportation (7). It is also one of the major entry ports for infectious pathogens in general, especially respiratory viruses, due to extensive travel of its resident population and millions of incoming visitors. Indeed, New York emerged as an early epicenter of the coronavirus disease 2019 (COVID-19) pandemic (8, 9) after sporadic introductions of SARS-CoV-2 in the spring of 2020 (10). During the first 3 months of the pandemic, more than 200,000 people living in New York City were infected, 26% of whom required hospitalization (11). Nine percent of those hospitalized in NYC during March/April of 2020 died (11).

We leveraged our health system-wide precision surveillance infrastructure to map the changes in frequency of SARS-CoV-2 and eight common respiratory viruses (influenza A/B viruses, RSV, seasonal coronaviruses [sCoV], parainfluenza viruses [PIV], human metapneumovirus [HMPV], adenoviruses, and rhino/enteroviruses [RV/EVs]) over 312 weeks spanning 7 years (September 2019–August 2025). More than three million nucleic acid amplification tests, performed within the MSHS during this time were queried, yielding over 193,000 positive test results (Fig. S1).

**Fig 1:**
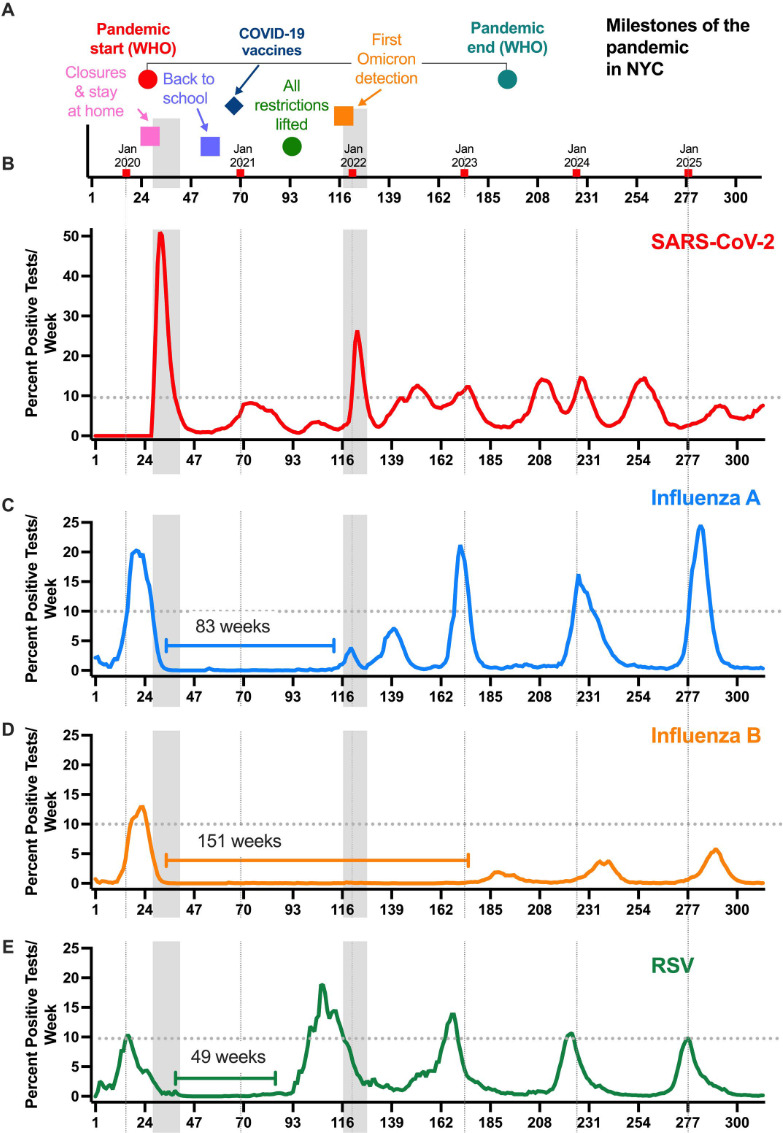
The circulation of influenza A virus, influenza B virus, and RSV was absent for extended durations following the initial wave of SARS-CoV-2 infections in March of 2020 in NYC. (**A**) Key dates specific to the COVID-19 pandemic in NYC are shown with the gray vertical bars identifying the first wave as well as the Omicron wave, both of which swept the metropolitan area. The beginning of each calendar year is indicated by vertical dotted lines in each panel. The dotted horizontal line in each plot indicates the 10% test positivity rate. Numbers of positive tests per week for SARS-CoV-2 (**B**; *N* positive tests: 123,675), influenza A virus (**C**; *N* positive tests: 31,131), influenza B virus (**D**; *N* positive tests: 6,982), and RSV (**E**; *N* positive tests: 14,467) are shown (312 weeks, 2019–2025). During this time, the test positivity for SARS-CoV-2 never dipped below 0.5%. The length of time a given virus remained undetected (under 0.5% frequency for two consecutive weeks) is shown by the bracket line. Frequency counts in the panels are smoothed using an exponentially weighted window with a span of 4.

**Fig 2:**
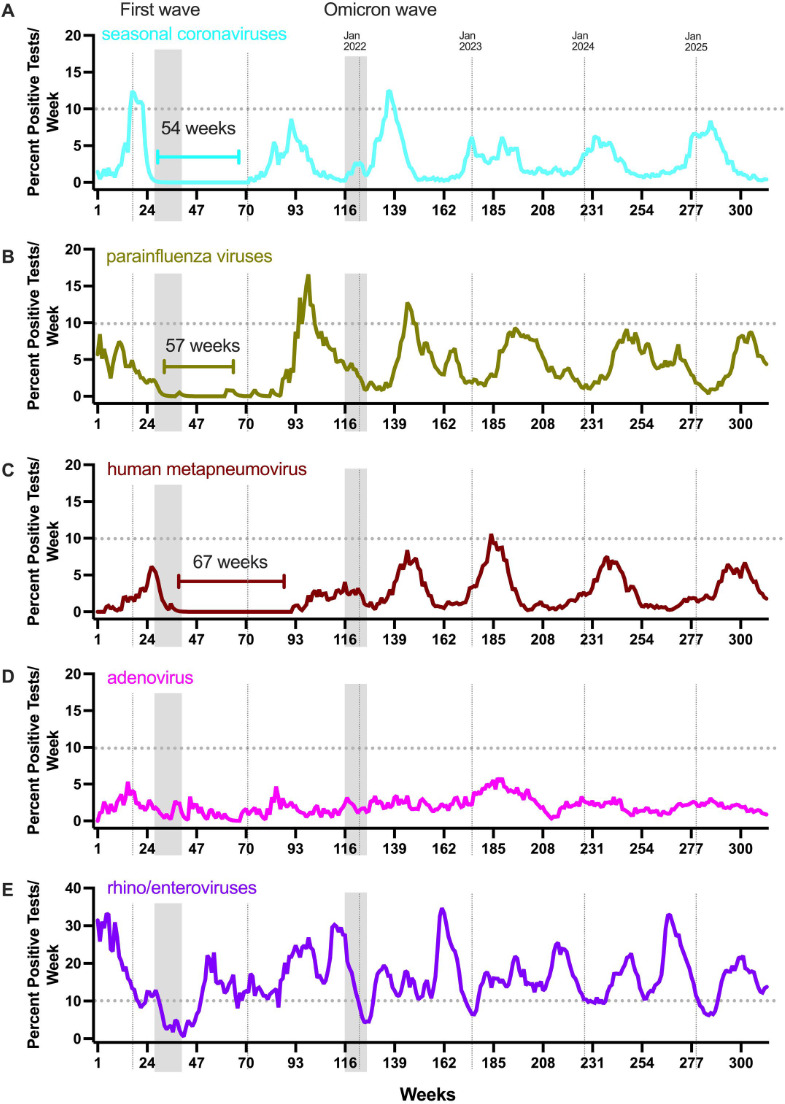
The circulation of seasonal coronaviruses (sCoVs), parainfluenza viruses (PIVs), and human metapneumovirus (HMPV) was absent for extended durations following the initial wave of SARS-CoV-2 infections in March of 2020 in NYC. The gray vertical bars identify the first wave as well as the Omicron wave (same as in Fig. 1), and the dotted vertical lines identify the beginning of each calendar year. The dotted horizontal line in each plot indicates the 10% test positivity rate. Numbers of positive tests per week for sCoV (**A**; *N* positive tests: 2,377), PIV (**B**; *N* positive tests: 3,599), HMPV (**C**; *N* positive tests: 2,398), adenoviruses (**D**; *N* positive tests: 1,825), and RH/EV (**E**; *N* positive tests: 13,650) are shown (Biofire Respiratory Panels 2.0 and 2.1, 312 weeks, 2019–2025). The length of time a given virus remained undetected (under 0.5% frequency for two consecutive weeks) is shown by the bracket line. Frequency counts in the panels are smoothed using an exponentially weighted window with a span of 4.

The epidemiological data for influenza A/B viruses have been robust and predictable over the last several decades until the outbreak of SARS-CoV-2 (1). The seasonality of SARS-CoV-2 initially differed drastically from that of other RNA-containing respiratory viruses: SARS-CoV-2 circulated year-round with notable peaks (waves) both in winter as well as summer starting in 2020 (Fig. 1). After in-house molecular testing for SARS-CoV-2 became available in the MSHS (mid-March 2020 corresponding to week 28 in the data set shown in Fig. 1 and 2), there was not a single week without positive SARS-CoV-2 test results for the next 5.5 years (284 weeks, March 2020–August 2025).

The first SARS-CoV-2 wave in NYC coincided with rapid decreases followed by a prolonged absence of influenza A virus (83 weeks), influenza B virus (151 weeks), RSV (49 weeks), sCoV (52 weeks), PIV (57 weeks), and HMPV (67 weeks) (Fig. 1 and 2). We refer to this disruption of seasonal patterns as the “pandemic gap” of respiratory viruses. Of note, the pandemic gap was specific for enveloped respiratory viruses since a near-continuous circulation was observed for adenoviruses (non-enveloped DNA viruses) and RV/EV (non-enveloped RNA viruses) (Fig. 2). These distinctive transmission patterns could be due to prolonged survival on fomites or result from differences in susceptibility to environmental factors such as temperature and humidity (2).

When the circulation of these viruses resumed, the timing was offset by months. Remarkably, the Yamagata lineage of influenza B viruses never reemerged, while viral strains of the Victoria lineage reappeared only in the winter of 2023. The winter season of 2024–2025 marked a notable return to pre-pandemic patterns. Despite the high-severity influenza season (12), SARS-CoV-2 infections remained below 10% test positivity through 2025 (Fig. 1). In addition, surges for sCoV, HMPV, and RV/EV were noted in the 2024–2025 fall/winter months. For the latter viral pathogens, we lack solid pre-pandemic comparisons as the multiplex respiratory diagnostic platforms that included these tests (e.g., BioFire Respiratory Panel) were infrequently ordered at MSHS prior to 2020.

This study provides data spanning the COVID-19 pandemic tracking the dynamics of respiratory viruses. It was shown previously that large health care systems such as MSHS are well suited to determine the prevalence of SARS-CoV-2 in a manner representative of large metropolitan areas (13, 14). It is likely that the 2- to 3-year absence of influenza A viruses (late winter 2020 to early winter 2022) and influenza B viruses (late winter 2020 to late winter of 2023) will shape the immune imprinting of children born in this period (15). Future studies will provide insights into the impact of the pandemic gap on the susceptibility to respiratory viral pathogens.

The pandemic gap of respiratory viruses, described here, coincided with the appearance of SARS-CoV-2. One explanation for this phenomenon is that public health interventions aimed at breaking the chain of transmission, such as reduced person-to-person contacts (e.g., social distancing and closing of businesses and schools) and physical barriers (the wearing of face masks) contributed to the prolonged absence of these different respiratory pathogens (6). Whether any of these measures alone was a major factor in interrupting the circulation of respiratory viruses or whether several conditions synergistically contributed to this epidemiological pattern remains unclear at present.

Another contributing factor to the pandemic gap of respiratory viruses observed could be the presence of a high proportion of subclinical SARS-CoV-2 infections. People with asymptomatic infections may have exhibited an interferon-mediated antiviral state in their respiratory tracts, thereby lowering the reproduction number of other respiratory viruses in the broader population. In this respect, we note that the initial Omicron wave in NYC (December 2021–February 2022, weeks 122–127 in Fig. 1 and 2) also coincided with a temporary reduction in detection of other respiratory viruses. This reduction was most pronounced for influenza A infections, resulting in a highly atypical influenza A virus bimodal winter peak (Fig. 1).

Significant reductions in the circulation of common respiratory viruses have also been seen in other parts of the world during the COVID-19 pandemic (4, 6, 16, 17). In general, infectious diseases that are transmitted through the air were most severely affected by the pandemic (varicella, pertussis, mumps, invasive *H. influenzae*, and influenza A/B viruses [17]). While non-pharmaceutical interventions during the SARS-CoV-2 pandemic may have interrupted the circulation of respiratory viruses, the induction of antiviral interferons may have contributed to the disappearance of respiratory viruses. The latter phenomenon may be part of what is known as trained immunity (18). Infection by certain viruses and bacteria can lead to a long-term increase in the activation of innate immune cells, resulting in protection against heterologous pathogens. Specifically, immunization with Bacillus Calmette-Guerin, measles virus (in the measles, mumps, rubella vaccine), or the poliovirus vaccine has been demonstrated to lead to immunity against unrelated pathogens (19–21). The disappearance of unrelated enveloped respiratory viruses during the initial overwhelming wave of SARS-CoV-2 may, thus, be due to the role of trained immunity. Further studies will be necessary to identify the precise mechanism by which this immune protection is induced and which cell types are involved.

This study has several limitations. First, the initial numbers for SARS-CoV-2 testing are likely an underestimate of the total number of positive cases seen in the system, given the limited availability of diagnostic tests early in the pandemic. Although our pathogen surveillance framework is focused on the MSHS, which comprises several hospitals serving the NY metropolitan area, the data also reflect the impact of successive epidemiological waves of SARS-CoV-2 and the widespread public health interventions such as masking, social distancing, lockdowns, and vaccination rollout in the highly populated NYC area during the first years of the pandemic. Given that the scope and timing of these measures varied by region and country, our findings may not be representative of what happened in other regions of the United States or the world at large.

## SUPPLEMENTAL MATERIAL

Supplemental Material mbio.03376-25-s0001.docx Supplemental methods and Fig. S1.

ASM does not own the copyrights to Supplemental Material that may be linked to, or accessed through, an article. The authors have granted ASM a non-exclusive, world-wide license to publish the Supplemental Material files. Please contact the corresponding author directly for reuse.
