# Supplementary material for: The pandemic gap of respiratory viruses during the COVID-19 pandemic
Source: mBio. 2025 Dec 10;17(1):e03376-25. doi: 10.1128/mbio.03376-25 (PMC12802283; doi:10.1128/mbio.03376-25)
Supplement: Supplemental Material — Supplemental methods and Fig. S1. [file mbio.03376-25-s0001.docx]

**Supplemental Online Material**

**In-depth methodological descriptions**

**Nucleic acid amplification tests (NAAT):** Results from over 3,700,000 diagnostic tests were included in this study. Several different test platforms have been utilized over the seven years included in this study. During the pandemic years (2020-2023), several tests were used, often dictated by supply chain interruptions. No substantial changes in NAAT sensitivity and specificity were observed during the study period.

*SARS-CoV-2 testing platforms.* The following 11 different assays were used throughout the reporting period: ePlex® SARS-C0V-2 Test EUA [GenMark Diagnostics, Inc]; SARS-CoV-2 PCR LDT/EUA [In-house based on CDC RT qPCR primers/probe]; cobas® SARS-CoV-2 Qualitative PCR EUA [Roche]; Xpert® Xpress SARS-CoV-2 real-time RT-PCR EUA [Cepheid]; Simplexa™ COVID-19 Direct, real time RT-PCR, EUA [DiaSorin Molecular]; Aptima® SARS-CoV-2 Assay, transcription-mediated amplification/dual kinetic assay, EUA [Hologic]; BioFire® COVID-19 Test, nested multiplexed real-time RT-PCR EUA [BioFire Defense LLC]; MassARRAY® SARS-CoV-2 Panel EUA (Agena Bioscience]; SARS-CoV-2 RNA STAR Complete EUA [lumiraDx]; PerkinElmer® New Coronavirus Nucleic Acid Detection Kit real time RT-PCR IVD-EUA; Xpert® Xpress CoV-2 PLUS real-time RT-PCR EUA [Cepheid].

*Multiplex respiratory virus targets.* More than 1,940,000 multiplex assays were used throughout the reporting period.

Influenza A, influenza B, RSV: Two different tests were used prior to the SARS-CoV-2 pandemic: Xpert® Xpress Flu/RSV multiplexed real-time RT-PCR EUA [Cepheid] and cobas® liat PCR EUA [Roche].

SARS-CoV-2, influenza A, influenza B: Starting December 2020, the cobas® liat SARS-CoV2 & Influenza A/B PCR EUA [Roche] became available.

SARS-CoV-2, influenza A, influenza B, RSV: Xpert® Xpress CoV-2/Flu/RSV, multiplexed real-time RT-PCR EUA [Cepheid]; Xpert® Xpress CoV-2/Flu/RSV *PLUS*, multiplexed real-time RT-PCR EUA [Cepheid].

Syndromic Respiratory panels: The BioFire® Respiratory Panel 2 nested multiplexed real-time RT-PCR [bioMérieux] was used before the pandemic and throughout the first year of the pandemic. The BioFire® Respiratory Panel 2.1 nested multiplexed real-time RT-PCR EUA/De Novo Authorized [bioMérieux], which supports detection of SARS-CoV-2, was implemented in December 2020. Both multiplex panels detect influenza A virus, influenza B virus, RSV, seasonal coronaviruses (OC43, HKU1, 229E and NL63), parainfluenza viruses (types 1 to 4), human metapneumovirus, and human rhinovirus/ enterovirus.

**The Mount Sinai Pathogen Surveillance Program (MS-PSP)**: The MS-PSP is a comprehensive, system-wide MSHS research initiative focused on detecting, monitoring, and ultimately preventing infectious diseases (Mount Sinai Hospital IRB approved HS#13-00981). Supported by a multidisciplinary team spanning microbiology, genomics, data science, and clinical medicine, the program integrates de-identified medical records with pathogen genomic data from residual clinical biospecimens collected across MSHS’s extensive hospital and clinic network. This infrastructure supports the tracking of pathogen frequency not only in a retrospective manner but also in near real time.

**Definition of the virus specific gap periods**: We selected conservative metrics for the definition of the gap periods. For SARS-CoV-2, influenza A/B and RSV, a relative test positivity under or equal to 0.5% was used to define virus specific gap periods. The beginning and end of the gap was defined by two consecutive weeks with >0.5% test positivity.

Throughout the calendar year, the overall diagnostic test volume for respiratory pathogen fluctuates (**Supplemental Figure 1**). In this study, a 0.5% relative test positivity was used as a threshold for defining the pandemic gap. A minimum denominator of 50 assays is required to ascertain that threshold (e.g., 0.4% positivity reflects two positive tests out of 50 ordered per week). Between weeks 40 and 52 (June to September 2020), the number of tests ordered for influenza A/B and RSV was below 100 per week. During this period, no or only a single positive result was recorded. Testing practices during the first six months of the pandemic defaulted to SARS-CoV-2 testing, followed by influenza A/B and RSV testing in case of a negative SARS-CoV-2 test result.

The overall test numbers for the BioFire® Respiratory Panel were consistently equal or below 50 tests per week from May to September 2020 (week 36 to 53). From September 2020 to March 2021 (week 55 to 86), there were intermittingly weeks with 50 or fewer BioFire® Respiratory Panel tests ordered. We used no or only one positive test result per week as a hallmark of the gap periods for sCoVs, PIV, HMPV, adenovirus and RH/EV. Two or more positive tests in two consecutive weeks marked the end of the gap for a specific virus.

**Suppl. Figure 1**

**

**

**Suppl. Figure 1**: Overview of the more than three million molecular tests ordered within MSHS for respiratory viruses (September 2019-August 2025). Note the different ranges of the y-axis for SARS-CoV-2 (A), influenza viruses/RSV (B) and the BioFire® Respiratory panels (C). The grey vertical bars identify the first wave as well as the Omicron wave.

Panel B1 provides a zoom-in for influenza A, influenza B and RSV test numbers during the period between week 24 and 70.

Panel C1 provides a zoom-in of the BioFire® Respiratory Panel test numbers during the period between weeks 24 and 116.
